# Alloxan-Induced Diabetes Causes Morphological and Ultrastructural Changes in Rat Liver that Resemble the Natural History of Chronic Fatty Liver Disease in Humans

**DOI:** 10.1155/2015/494578

**Published:** 2015-02-19

**Authors:** Amanda Natália Lucchesi, Lucas Langoni Cassettari, César Tadeu Spadella

**Affiliations:** ^1^Graduate Program in General Basis of Surgery, Faculty of Medicine, São Paulo State University (UNESP), 18618-970 Botucatu, SP, Brazil; ^2^Faculty of Medicine, São Paulo State University (UNESP), 18618-970 Botucatu, SP, Brazil; ^3^Department of Surgery and Orthopedics, Faculty of Medicine, São Paulo State University (UNESP), 18618-970 Botucatu, SP, Brazil

## Abstract

*Purpose*. This study evaluated the long-term effects of alloxan-induced diabetes in rat liver.* Methods*. Thirty nondiabetic control rats (NC) and 30 untreated diabetic (UD) rats were divided into three subgroups sacrificed after 6, 14, or 26 weeks. Clinical and laboratory parameters were assessed. Fresh liver weight and its relationship with body weight were obtained, and liver tissue was analyzed.* Results*. UD rats showed sustained hyperglycemia, high glycosylated hemoglobin, and low plasma insulin. High serum levels of AST and ALT were observed in UD rats after 2 weeks, but only ALT remained elevated throughout the experiment. Fresh liver weight was equal between NC and UD rats, but the fresh liver weight/body weight ratio was significantly higher in UD rats after 14 and 26 weeks. UD rats showed liver morphological changes characterized by hepatic sinusoidal enlargement and micro- and macrovesicular hepatocyte fatty degeneration with progressive liver structure loss, steatohepatitis, and periportal fibrosis. Ultrastructural changes of hepatocytes, such as a decrease in the number of intracytoplasmic organelles and degeneration of mitochondria, rough endoplasmic reticulum, and nuclei, were also observed.* Conclusion*. Alloxan-induced diabetes triggered liver morphological and ultrastructural changes that closely resembled human disease, ranging from steatosis to steatohepatitis and liver fibrosis.

## 1. Introduction

An association between type 2 diabetes mellitus (T2DM) and nonalcoholic fatty liver disease (NAFLD), which describes a wide spectrum of liver disorders from steatosis to cirrhosis, has long been recognized [1–3]. Much attention was given to this entity in patients with T2DM, but little is known about the evolution of liver injury in patients with type 1 diabetes mellitus (T1DM).

Animal models of T1DM using chemical induction by alloxan (ALX) or streptozotocin (SZ) could provide valuable information about the natural history of NAFLD and improve our understanding of the mechanisms responsible for this condition. However, few long-term studies of liver injury were well documented [4–6].

In addition, due to the short follow-up of animals, much of the published experimental research on T1DM could not define clearly whether observed histopathological liver injuries were the result of toxic effects of ALX or SZ or linked to the drug-induced diabetic hyperglycemic state [7–13].

Therefore, this study evaluated whether T1DM induced by ALX could cause morphological and ultrastructural changes in rat liver to characterize the effects of this drug and diabetes for a longer follow-up period. We hope that our results can have reproduced a suitable experimental model for the investigation of diabetic chronic liver disease.

## 2. Methods

### 2.1. Animals and Diabetes Induction

The Animal Experimentation Ethics Committee of our institution approved the use of laboratory animals. Sixty male Wistar rats, approximately 250 g, were used in this experiment. Rats were conditioned in polyurethane boxes, placed in an air-conditioned environment at 25°C with controlled lighting and exhaust, and received standard balanced chow for rodents and water* ad libitum*.

Diabetes was induced using intravenous alloxan (Sigma Co, USA) at a dose of 42 mg/kg of body weight into tail veins. Only animals with clinical signs of severe diabetes and fasting glucose ≥ 250 mg/dL in two successive determinations (1 and 2 weeks after diabetes induction) were used. Diabetic animals that died during the postinduction period or at follow-up were replaced to avoid compromising the final number of rats in the sample.

Animals were randomly assigned to 2 experimental groups: NC: 30 nondiabetic control rats; UD: 30 diabetic rats without hyperglycemia treatment. Each group was further divided into 3 subgroups of 10 rats each to be sacrificed after 6, 14, and 26 weeks of follow-up or diabetes.

### 2.2. Analyzed Parameters

Clinical (body weight, water intake, food intake, and urine output) and laboratory (blood glucose, urine glucose, glycosylated hemoglobin, plasma insulin, bilirubin, AST, ALT, alkaline phosphatase, *γ*-glutamyl transferase, total cholesterol, triglycerides, and high and low density lipoproteins) parameters were analyzed after 2 weeks of follow-up or diabetes and at each time point of sacrifice. Clinical parameters were obtained using individual metabolic cages. Blood biochemical tests were performed in a chemical dry system using Vitros (Johnson and Johnson, USA). Glycosylated hemoglobin was measured using electrophoresis in agarose gel (Sebia, France), and insulin was measured using radioimmunoassay (Diagnostic Products Corporation, USA). Fresh liver weight and its relationship with body weight were obtained at sacrifice, and liver tissue fragments were analyzed using optical and transmission electron microscopy.

### 2.3. Technical Procedures

All invasive procedures on animals were performed under general anesthesia using intramuscular ketamine (100 mg/kg of body weight) + xylazine (50 mg/kg of body weight) (Rhobifarma Ind. Farmacêutica Ltda, Brazil). Blood samples were obtained from the tail vein at follow-up and via cardiac puncture with the chest open at sacrifice. The entire liver was removed, rinsed, and weighed on an analytical balance after exsanguination. Segments representing the entire organ were randomly taken from 15 rats in each experimental group (5 animals per subgroup of sacrifice) and cut, prefixed in buffered 10% formalin, processed in paraffin blocks, and sectioned at 3–5 *μ*m for histological analyses. Two liver tissue blocks were prepared from each rat. Two histological slides from each block were stained with hematoxylin and eosin (HE), and 1 slide was stained with red picrosirius. Thirty slides (each containing two sections of liver) were analyzed by rat subgroup, for a total of 90 slides for each experimental group (60 HE; 30 red picrosirius). Two different investigators who were unaware of the experimental group examined all slides using light microscopy. All analyses used a descriptive qualitative method and were supervised by an experienced pathologist. Small pieces of liver were prefixed in 2.5% glutaraldehyde, washed in a cacodylate buffer solution and postfixed in 1% osmium tetroxide for ultrastructural analyses. Specimens were dehydrated using ascending grades of alcohols and embedded in an epon-araldite mixture. Ultrathin sections stained with uranyl-acetate and lead citrate were examined under a transmission electron microscope (Phillips–CM 100). Livers from 9 rats of each experimental group were randomly collected for these analyses (3 per sacrifice subgroup). One block of liver tissue was prepared from each rat, and 10 electron micrographs of hepatocytes were obtained at magnifications between 1,200x and 4,800x. A total of 30 electron micrographs were examined per subgroup of rats, or 90 per experimental group. All analyses used a descriptive method and were supervised by an experienced pathologist.

### 2.4. Statistical Analysis

Analyses of clinical and laboratory variables and fresh liver weight and its relationship with body weight in the 2 experimental groups at 4 or 3 time points of evaluation or sacrifice, respectively, were conducted using analysis of variance, in a completely randomized design, followed by Tukey's multiple comparisons test for homogeneous or parametric variables or Mann-Whitney nonparametric analysis for variables showing results with a heterogeneous distribution. All statistical discussions in the study were performed at a level of significance of 5% (*P* < 0.05).

## 3. Results

### 3.1. Clinical and Laboratory Findings

**Figure 1:**
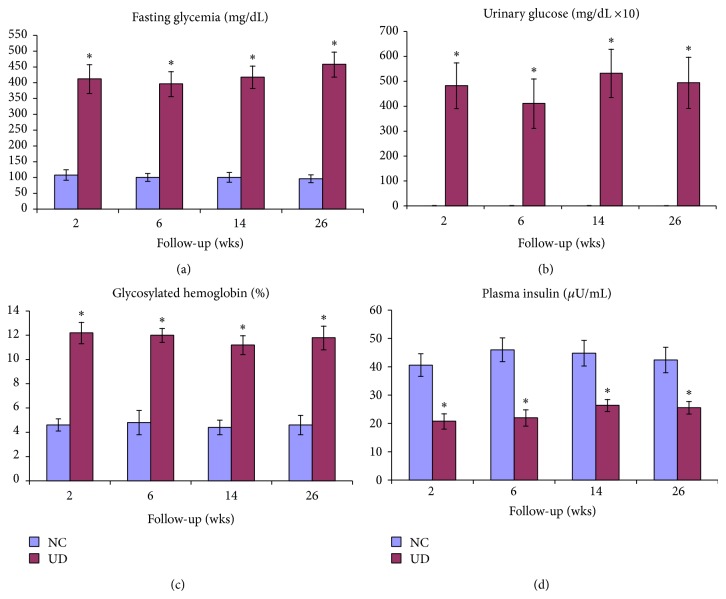
Mean ± SD of fasting glycemia (a), urinary glucose (b), glycosylated hemoglobin (c), and plasma insulin (d) in nondiabetic control (NC) and untreated diabetic animals (UD) during follow-up time, given in weeks.

NC rats showed clinical and laboratory parameters that were compatible with normal animals of the same strain at all evaluation points of the experiment. In contrast, UD rats without hyperglycemia treatment progressed to a severe loss of body weight and significant increase in water and food intake and urine output compared to NC rats (*P* < 0.001). Blood glucose, urine glucose, and glycosylated hemoglobin were persistently elevated in UD rats, and plasma insulin values were significantly lower (Figure 1). High serum levels of AST and ALT were observed in UD rats after 2 weeks of diabetes. AST values returned to normal levels after 6 weeks, but ALT levels remained significantly elevated until the end of the experiment. There were no significant differences in other liver function tests or the concentration of blood lipids and low and high density lipoproteins (HDL; LDL) during the study (Tables 1 and 2).

### 3.2. Fresh Liver Weight and Fresh Liver Weight/Body Weight Ratio

Fresh liver weight did not differ between UD and NC rats at any time point of sacrifice, but the fresh liver weight/body weight* ratio* was significantly higher in UD rats after 14 and 26 wks (Table 3).

### 3.3. Morphological Findings of Light Microscopy

**Figure 2:**
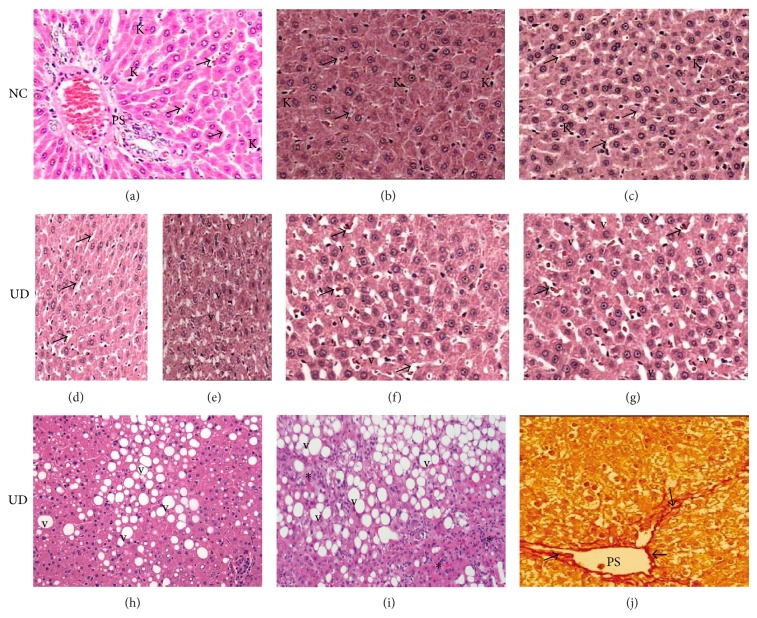
(a), (b), and (c): histological pattern of liver from nondiabetic control rats (NC) showing normal-appearing of hepatocytes, portal space (PS), sinusoids (arrows), and Kuppfer cells (K) at 6, 14, and 26 weeks of follow-up, respectively. Focal area of mild steatosis (∗) is observed in NC rats sacrificed at 26 weeks. (d) and (e): liver from untreated diabetic rats (UD) sacrificed at 6 weeks showing the onset of sinusoidal enlargement (arrows) and small amount of fatty vacuoles (v), respectively. (f) and (g): liver from UD rats sacrificed at 14 and 26 weeks showing progressive worsening of sinusoidal enlargement (arrows) and liver fatty degeneration (v), respectively. Liver from UD rats at 26 weeks showing in (h) macrovesicular fatty degeneration (v); (i) interlobular mononuclear inflammatory infiltrate consistent with steatohepatitis (∗); (j) periportal (PS) fibrosis (arrows). H&E and red picrosirius (400x).

NC rats showed liver parenchyma with general structures preserved, including hepatic lobules with normal hepatocytes surrounded by sinusoids and distributed radially towards the centrilobular veins, and containing Kupffer and red blood cells in the capillary lumen. Portal spaces were also normal, with no observed inflammatory infiltration, fatty degeneration or abnormal distribution of fibroblasts or collagen in rats sacrificed at 6 and 14 weeks of follow-up. Only mild focal microvesicular fatty degeneration was observed in the livers of NC animals sacrificed at 26 weeks of follow-up, but no other abnormalities were observed. In contrast, UD rats presented morphological changes in the liver that had a close temporal relationship with diabetic follow-up. These changes were characterized by hepatocytes that contained focal or generalized fatty vacuoles and micro- or macrovesicular features that were associated with the presence of dilated sinusoids and a progressive loss of general organ structure. Inflammatory changes consistent with steatohepatitis, which were represented by mononuclear inflammatory infiltrates of moderate intensity, were observed in the interlobular and periportal spaces in 40% of analyzed liver histological sections from animals sacrificed at 26 weeks of diabetic follow-up. Mild hepatic fibrosis surrounding the portal vessels was also observed in 20% of liver histological sections stained with red picrosirius in this subgroup of animals. Suggestive histological evidence of cirrhosis or hepatocellular carcinoma was not observed.  Figure 2 illustrates the morphological findings of the two experimental groups.

### 3.4. Ultrastructural Findings of Transmission Electron Microscopy

**Figure 3:**
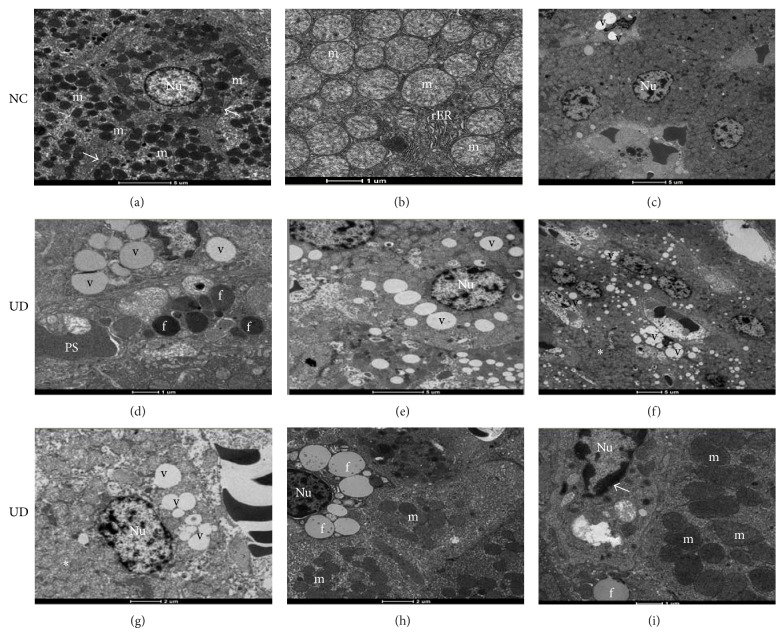
Electron micrographs of nondiabetic control rats (NC) sacrificed at 6, 14, and 26 weeks, respectively, showing (a) normal-appearing of hepatocytes, nucleus (Nu), mitochondria (m) and Disse's space (arrows); (b) detail of mitochondria (m) and rough endoplasmic reticulum (rER) with their preserved structures; (c) focal area of lipid vacuoles (v) in NC rat sacrificed at 26 weeks (scale bars: (a), (c): 1,900x; (b): 4,800x). (d), (e), and (f): hepatocytes of untreated diabetic rats (UD) sacrificed at 6, 14, and 26 weeks, respectively, showing (d) hepatocyte showing moderate amount of lipid vacuoles (v) and hepatic lipid droplets (f); (e) worsening of liver fatty degeneration (v); (f) poor organization of the organelles within the cytoplasm with disappearance of the Disse's space and rER (∗) with diffuse liver fatty degeneration (v) (scale bars: (d): 4,800x; (e), (f): 1,900x). (g), (h), and (i): hepatocytes of UD rats sacrificed at 26 weeks showing: (g) intense disorganization of cell ultrastructure (∗); (h) scarce amount of mitochondria (m) within the cytoplasm; (i) mitochondria appear larger, less electron-dense with less distinct cristae (m). Note the condensation of nuclear chromatin (arrow) (scale bars: (g), (h), (i): 4,800x).

Ultrastructural findings of the liver showed significant differences between nondiabetic and diabetic animals. Similarly, the severity of lesions in UD rats was closely related to the time of diabetic follow-up; lesions were visibly more severe at 26 weeks. Hepatocytes from these animals showed a progressive disruption of structural architecture that was characterized by an apparent decrease in the number of intracytoplasmic organelles and associated with fat deposition and liver vacuolization that was similar to micro- and macrovesicular steatosis. Degenerative changes in mitochondria, rough endoplasmic reticulum (rER) and nuclei were also observed in hepatocytes from UD rats sacrificed at 26 weeks of follow-up. These changes were characterized by swollen mitochondria and an apparent decrease in the number of mitochondrial cristae and cisterns of the rER. Condensation of nuclear heterochromatin was also observed.  Figure 3 illustrates the ultrastructural findings.

## 4. Discussion

Clinical and experimental evidence suggests that diabetes mellitus (DM) affects the liver in addition to blood vessels, kidneys, retina and nerves [14–17]. However, the recognition of DM as the primary cause of chronic liver disease is neglected in medical practice because of the wide variety of clinical, metabolic and hormonal conditions that can lead to NAFLD, such as obesity, malnutrition, intestinal malabsorption, dyslipidemia, thyroid disorders, and metabolic syndrome [18].

This study demonstrated that rats with T1DM induced by intravenous ALX presented biochemical changes in blood and morphological and ultrastructural lesions in the liver that largely resembled chronic liver disease in humans. Liver changes ranged from the fatty degeneration of liver cells to steatohepatitis and periportal fibrosis.

Typically, the observed lesions in the livers of diabetic animals in this study reached all structures of the organ, including both portal areas and sinusoids, and hepatocytes, nuclei, and intracytoplasmic organelles, including a progressive enlargement of sinusoids, micro- and macrovesicular fatty degeneration, steatohepatitis and periportal fibrosis. Further, ultrastructural changes in hepatocytes, particularly the mitochondria, the rough endoplasmic reticulum (rER) and cell nuclei, were also observed.

However, whether the observed histopathological changes in the livers of ALX-induced diabetic rats were due to the toxic action of the drug or the diabetic state was not certain. ALX and streptozotocin (SZ) exert a toxic effect on pancreatic beta cells, which causes T1DM, but this effect extends to the kidneys and livers of animals of several species [19, 20]. However, the systemic toxicity of these drugs is closely related to species, age and body weight of the animals used and the hydration status, route of administration, infusion rate and duration of fasting for drug administration [21].

Systemic toxic actions of ALX and SZ are commonly observed during the first 2 weeks after diabetes induction. ALX displays a particularly narrow margin of safety between the diabetogenic and lethal dose, and deaths commonly occur as a result of diabetic ketoacidosis and/or kidney or liver failure [21]. These facts highlight the importance of performing experimental observations of the liver after the acute or subacute phases of ALX action because the real cause of the observed changes can be identified with more clearly after these periods.

Goel [22] studied ALX-induced diabetic fish during the first 100 h after drug administration and observed submassive necrosis of the liver in these animals. This author postulated that ALX altered the regular pathways of cellular metabolism, including the inactivation of certain enzymes, which led to liver damage and death. These findings were described previously, but these effects were not observed in the liver of animals in this study, which suggests that focal or diffuse hepatic necrosis is only observed in animals that are sacrificed or die during an early phase. Kume et al. [8] also reported acute morphological changes in the liver of mice treated with SZ, including hepatocyte hypertrophy, an increased number of intracytoplasmic acidophilus granules, and bile duct hyperplasia, which were also not observed in the livers of animals in this study. However, the results of these researchers suggest that the liver morphological alterations that are observed during the early stages of treatment with ALX or SZ may be more related to the toxic action of these drugs than to the effects of DM.

Voss et al. [23] showed that SZ had harmful effect on rat livers, even in the absence of a hyperglycemic state, which was revealed by a significant increase in blood levels of ALT. However, these authors noted that this effect was short-lived (10 days) and that only in animals which developed hyperglycemia the ALT levels remained elevated 30 days after diabetes induction. This finding was also observed in alloxan-induced diabetic animals in this study; blood levels of AST and ALT were elevated in the first 2 weeks after treatment, but only ALT remained significantly elevated at 6, 14 and 26 weeks after diabetes induction. In contrast to the data described by some authors [8, 9], we did not observe significant changes in other liver function tests, including liver enzymes of excretion, cholesterol, triglycerides, and low and high density lipoproteins at any time points of our experiment.

Kozyritskij and Minchenko [4] also observed significant ultrastructural changes in hepatocytes of alloxan-induced diabetic rats after a 12-week period, which was demonstrated by an increase in mitochondrial size, reduction in the development of the rER and little proliferation of smooth endoplasmic reticulum elements. Surprisingly, the administration of insulin and the restoration of normal blood glucose levels reversed all of the ultrastructural changes in the livers of these animals. This finding demonstrated that the observed hepatic lesions in rats, especially after the 2nd week of diabetes, were more related to insulin deficiency and the hyperglycemic state than to the toxic effects of this drug on the liver. Subsequent studies confirmed this hypothesis by demonstrating that the effective control of abnormal blood glucose levels in diabetic animals relieves and/or restores a normal liver histological pattern [9, 11–13, 24].

Notably, some liver lesions caused by the toxic action of ALX or SZ during the acute and subacute phases of treatment are also observed during chronic stages of diabetes induction, which suggests that these lesions may result from separate effects of these chemicals or chronic hyperglycemia and the combined action of these factors. Laguens et al. [7] suggested this hypothesis by noting that a dilation of cisterns and degranulation of the rER surface, mitochondrial swelling and a loss of mitochondrial cristae were observed in hepatocytes of C3H-s mice 21 days after SZ treatment both in animals that developed diabetes and in those that did not develop the disease. Mahmoud and Al-Salahy [9] demonstrated that hyperglycemia plays an important role in the genesis of liver injury by inducing an enlargement of sinusoids, dilation of cisterns and a loss of rER ribosomes in the hepatocytes of fish treated with repeated doses of glucose and fish that received ALX only. These authors also showed that normalization of blood glucose levels, using an aqueous suspension of lupine seeds, restored most histological and ultrastructural lesions in the livers of these animals.

The severity of the histopathological lesions in the livers of animals in this study maintained a close relationship with the time course of diabetes; severity increased with the duration of the experimental follow-up, especially in diabetic animals after 26 weeks of an uncontrolled hyperglycemic state. Enlargement of sinusoids and focal microvesicular fatty degeneration with vacuolization of liver cells was observed as early as the 6th week of diabetes. These initial lesions gradually spread to the rest of the liver parenchyma in the 14th week, and permeated the entire organ in the 26th week of diabetes, when the liver characteristically showed diffuse macrovesicular fatty degeneration that was sometimes accompanied by interstitial mononuclear inflammatory infiltrates (steatohepatitis) and mild periportal fibrosis. However, the latter two changes were only found in animals sacrificed at 26 weeks of diabetes, which suggests that the evolution of liver fatty degeneration to steatohepatitis and fibrosis is dependent on the chronic exposure of liver cells to high concentrations of blood glucose. Moreover, liver cirrhosis and hepatocellular carcinoma were not observed in histological sections of livers from animals in this study at any time of sacrifice, which suggests that injuries of this nature may be observed in studies with longer follow-up periods.

Ultrastructural progressive impairment was also seen in the livers of diabetic animals in this study. These changes were characterized by disorganization of the liver cell ultrastructure, loss of intracytoplasmic organelles, micro- and macrovesicular fatty degeneration, and qualitative and specific changes of intracytoplasmic organelles and nuclei, which manifest as a lack of rER development, mitochondrial swelling, an apparent decrease of mitochondrial crests, and an abnormal distribution of heterochromatin granules.

Welt et al. [5] described similar findings using morphological and morphometric analyses of liver tissue from Wistar rats exposed to SZ-induced experimental diabetes or submitted to acute hypoxia over a period of 16 weeks. These authors observed an increase in the size of hepatocytes and their nuclei, a decreased nucleus/cytoplasm ratio and hepatic glycogen content, a deficient number of rER cisterns, and the presence of numerous fat vacuoles in liver cells (“Ito cells”) with an increased density of types I, III and VI collagens around the portal vessels and within the space of Disse, which denoted hepatic fibrosis. Doi et al. [25] also indicated that liver cell nuclei were also affected by DM, having noticed hepatocyte nuclei duplicate and heterogeneity in form, size, and distribution of nuclear chromatin granules in addition to the increased area of hepatocytes.

Guven et al. [12] investigated the protective effects of melatonin on liver injury in SZ-induced diabetic rats over a period of 4 weeks, and they also observed sinusoid congestion, hydropic degeneration, focal necrosis and microvesicular fatty infiltration of hepatocytes that was associated with moderate periportal inflammation, an accumulation of a homogeneous substance in nuclear chromatin, a decreased number of intracytoplasmic organelles and mitochondrial degeneration. However, these authors found no macrovesicular fatty degeneration or hepatic fibrosis, which supports the chronic nature of these changes. Similar results were also observed by Adewole and Ojewole [13] while studying the effects of an herbal aqueous extract of* Artocarpus communis* on blood glucose and biochemical, morphological and ultrastructural changes in the liver of SZ-induced diabetic rats using the same time of experimental follow-up. In contrast, Remedio et al. [6] examined the benefits of exercise on rat liver morphology in T1DM induced by ALX for a period of 8 weeks and observed a remarkable increase in the number of mitochondria in the liver cells of sedentary diabetic animals compared to sedentary controls with no evidence of significant ultrastructural changes in these organelles.

There is a consensus that DM affects the liver, such as other organs, in the long-term. The pathways of liver injury in T2DM are relatively well known, but little is known about the pathophysiology of chronic liver disease in patients with T1DM.

Cellular oxidative stress (OS) plays an important role in the genesis and progression of chronic diabetic lesions on vessels, kidneys, retina, nerves, and likely the liver of diabetic humans and animals [14, 26–28]. Méndez-Sánchez et al. [29] demonstrated that the persistence of a diabetic condition and chronic stress in liver cells is one of the most important predictors for the development of cirrhosis in diabetic patients with NAFLD, particularly in obese patients. Haque and Sanyal [30] postulated that the increased oxidation of free fatty acids (FFAs) by the liver can generate reactive oxygen species (ROS), which induce lipid peroxidation and cause structural and functional changes in the cells and cell death. The body's antioxidant defenses neutralize the harmful effects of these substances under physiological conditions. However, this metabolic balance is broken under pathological conditions, such as in diabetes, which initiates oxidative stress. The maintenance of hepatic oxidative stress triggers adaptation responses to chronic stress in the liver, which include the activation and/or inhibition of several molecular sites that transduce and transcribe signals that regulate the biological cell cycle [31]. Changes of the biological cell cycle ultimately compromise replication ability and liver regeneration, which leads to apoptosis or cell death. However, an excessive amount of FFAs in the liver alone may induce apoptosis of hepatocytes, which is likely one mechanism of cellular injury that is commonly observed in patients with NAFLD [32].

A previous study in our laboratory [33] showed that T1DM changed the oxidative balance in the liver of ALX-induced diabetic rats over the long-term, which was characterized by a significant increase in ROS in liver tissue and markedly reduced defense antioxidants. These results suggest that changes in blood liver enzymes (AST and ALT) and the morphological and ultrastructural lesions found in the livers of animals in this study are closely correlated to DM-induced chronic stress in liver cells.

However, the molecular pathways by which chronic stress induces histopathological lesions in the liver in patients with T1DM are not clear because the mechanisms of hyperinsulinemia and hepatic and peripheral resistance to insulin are not usually the predominant mechanisms.

Regnell and Lernmark [16] suggested a role of three mechanisms, separately or in combination, in the pathogenesis of NAFLD in patients with T1DM: (1) atypical rates or malfunction of lipoproteins would create alterations in triglyceride secretion by liver cells and lead to an accumulation of fat in the liver; (2) activation of transcription factors of hepatic metabolism by hyperglycemia, such as sterol regulatory element-binding proteins (SREBPs) and the carbohydrate response element-binding protein (ChREBP), would promote hepatic lipogenesis; and (3) excess liver glucose transported by the glucose transporter 2 factor (GLUT2) would lead to intrahepatic fat synthesis.

Several quantitative and qualitative changes in lipoproteins are observed in T1DM patients, even patients with good metabolic control: increased proportion of cholesterol, triglycerides, and low and very low density lipoproteins (LDL; VLDL), changes in the composition of the peripheral layers of lipoproteins, apolipoprotein glycation, LDL oxidation, and the amount of small dense particles [34]. The lipoprotein-related changes, according to mentioned author, would change the function of these substances and lead to NAFLD. However, not all changes in lipoprotein metabolism are completely explained by hyperglycemia. A state of peripheral hyperinsulinemia caused by the constant administration of subcutaneous insulin in patients with T1DM would also play an important role in the genesis of these changes and the consequent accumulation of fat in the liver [34]. Conversely, it is possible that NAFLD is not a consequence of, but rather a contributor to the development of lipoprotein abnormalities observed in these patients [35]. However, in the present study, we did not observe any abnormalities in lipoprotein metabolism at any diabetic follow-up point despite sustained hyperglycemia.

SREBPs are factors utilized in the transcription of cell signals that directly activate the expression of over 30 genes related to the synthesis and release of cholesterol, fatty acids, triglycerides, and phospholipids [36].* In vitro* studies demonstrated that elevated concentrations of blood glucose increase the concentrations of SREBP-1c, which is a precursor of SREBPs, in the endoplasmic reticulum. SREBP-1c acts with ChREBP to increase the expression of lipogenic genes derived from pyruvate kinase (LPK) and the enzymes acetyl-CoA carboxylase (ACC) and fatty acid synthase (FAS) [37]. LPK catalyzes the conversion of phosphoenolpyruvate to pyruvate, which is the main source of ACC that is predominantly used in the synthesis of fatty acids [38]. FAS catalyzes the conversion of malonyl-CoA into long chain fatty acids [39]. Yamashita et al. [40] postulated that ChREBP can stimulate the transcription of genes that are responsible for LPK activation in response to hyperglycemia, even without the participation of insulin. Therefore, ChREBP is a very important contributory factor for fat accumulation in the liver of patients with T1DM [41].

The third mechanism that is involved in the pathogenesis of NAFLD is mediated by GLUT2 and regulated by SREBP1-c. GLUT2 is a facilitative glucose transporter on target cells that is primarily found in liver, kidney, gut and pancreatic *β*-cells [42]. Hepatic expression of GLUT2 in rats is stimulated by hyperglycemia and hypoinsulinemia [43]. Therefore, a large amount of glucose is transported to the liver during diabetic state, which generates an additional stock of this carbohydrate in hepatocytes that is rapidly diverted to the synthesis of fatty acids. These fatty acids are exported to the blood as lipoproteins and/or stored in the liver parenchyma in the form of triglycerides, which results in steatosis.

Regnell and Lernmark [16] also emphasize that insulin plays an important role in the genesis of steatosis in rodent hepatocytes because it modulates the flow of FFAs that is transported by protein pathways mediated by 2 and 5 FATPs (fatty acid transport proteins). The actions of 2 and 5 FATP are dose-dependent and bimodal, and they are mediated by insulin receptor substrate-2 (IRS-2) under low concentrations of insulin and insulin receptor substrate-1 (IRS-1) during states of hyperinsulinemia. These observations indicate that an ideal balance in circulating insulin levels is required for the regulation of fat metabolism in the liver. Therefore, deviations from this equilibrium, such as the lack of insulin production (T1DM) or an excess of insulin in the bloodstream because elevated peripheral resistance to insulin action (T2DM) or the inadequate administration of exogenous insulin, lead to fat accumulation in the liver [44].

However, whether NAFLD progresses to nonalcoholic steatohepatitis (NASH) and later to fibrosis and/or cirrhosis in patients with T1DM was not determined. Many postulated mechanisms for the genesis of these complications in T2DM patients, which are also present in patients with T1DM, may be suggested because a significant portion of these patients are also obese, and mechanisms of peripheral insulin resistance and endogenous or iatrogenic hyperinsulinemia are also observed [29].

Portincasa et al. [18] suggested that the onset and progression of NAFLD to NASH and liver cirrhosis involves two crucial factors: the first factor is related to the patients' risk factors, such as genetic defects and lifestyle, and the second factor is related to changes in intra- and extracellular molecular mechanisms that lead to liver damage, including FFA oxidation, ROS formation, lipid peroxidation, and proinflammatory cytokine release, which is responsible for inflammation, hepatocellular necrosis and fibrosis. Dowman et al. [45] noted that the accumulation of triglycerides in the liver increases the susceptibility of this organ to injury mediated by inflammatory cytokines/adipocytokines or hepatocellular oxidative stress, by favoring the development of NAFLD to NASH and/or fibrosis. These researchers suggest that FFAs also act directly on the liver to promote liver injury. These compounds can undergo *β*-oxidation, being esterified with glycerol to form triglycerides, which leads to fat accumulation in the liver. FFAs in the liver may also increase hepatic oxidative stress and/or activate various inflammatory pathways that contribute to the progression of NAFLD.

Roskams et al. [28] stated that dead liver cells in healthy individuals stimulate replication of mature hepatocytes to reconstruct the damaged liver tissue. However, hepatic oxidative stress in the presence of NAFLD can inhibit the replication of mature hepatocytes, which results in the expansion of hepatic progenitors (oval cells) that can differentiate into “hepatocyte-like cells” which are not hepatocytes. An increased amount of oval cells and “hepatocyte-like cells” is strongly correlated with the progression of NAFLD to hepatic fibrosis and hepatocellular carcinogenesis.

## 5. Conclusion

Alloxan diabetes induced biochemical changes in the blood and morphological and ultrastructural lesions in rat liver, ranging from steatosis to steatohepatitis and liver fibrosis, which closely resemble changes in the human liver. The severity of histopathological lesions in the liver maintained a close relationship with the timing of diabetes; lesions were more severe in the longer experimental follow-up periods, especially in diabetic animals after 26 weeks of uncontrolled hyperglycemia. This experimental model of diabetic chronic liver disease may serve as a basis for new investigations. The histological findings should be correlated with the experimental phase in which liver damage was observed.

## Figures and Tables

**Table 1:** Medians, maximum, and minimum values of parameters related to liver function of nondiabetic control and untreated diabetic rats during the various experimental follow-up periods.

Groups	Follow-up	Parameters				
		Bilirubin	AST	ALT	Alkaline Ph	*γ*-GT
		(mg/dL)	(U/L)	(U/L)	(U/L)	(U/L)
NC	2 weeks	0.42 (0.32–0.76)	30 (20–32)	36 (34–50)	68 (60–105)	84 (52–96)
UD		0.56 (0.38–0.80)	96^*^ (35–115)	87^*^ (42–108)	76 (66–102)	70 (58–105)

*P* value		0.6870	<0.001	<0.001	0.3860	0.4254

NC	6 weeks	0.50 (0.30–0.80)	26.5 (18–36)	41.0 (35–52)	79.5 (65–102)	70.5 (46–112)
UD		0.54 (0.38–0.75)	29.0 (18–36)	92.0^*^ (37–96)	85.0 (67–110)	80.5 (56–108)

*P* value		0.9705	0.6305	<0.001	0.4813	0.3930

NC	14 weeks	0.60 (0.55–0.74)	26.5 (22–30)	39.0 (28–50)	55.0 (48–72)	31.0 (22–45)
UD		0.70 (0.56–0.80)	28.5 (26–32)	80.0^*^ (66–97)	64.5 (50–80)	39.0 (32–50)

*P* value		0.3429	0.3229	<0.001	0.3362	0.2702

NC	26 weeks	0.39 (0.28–0.52)	42.0 (38–50)	43.0 (26–55)	55.0 (36–80)	40.5 (22–54)
UD		0.44 (0.30–0.60)	51.5 (40–56)	78.5^*^ (72–92)	67.5 (55–90)	53.0 (38–70)

*P* value		0.4857	0.2000	<0.001	0.3429	0.2000

NC: nondiabetic control; UD: untreated diabetic; AST: aspartate aminotransferase; ALT: alanine aminotransferase; Alkaline Ph: alkaline phosphatase; *γ*-GT: gamma glutamil transferase.

^*^NC < UD at 2, 6, 14, and 26 weeks of follow-up (Mann-Whitney test).

**Table 2:** Medians, maximum, and minimum values of parameters related to lipids profile of nondiabetic control and untreated diabetic rats during the various experimental follow-up periods.

Groups	Follow-up	Parameters			
		Total cholesterol	HDL cholesterol	LDL cholesterol	Triglycerides
		(mg/dL)	(mg/dL)	(mg/dL)	(mg/dL)
NC	2 weeks	176.0 (152–206)	48.0 (45–58)	22.0 (15–28)	118.0 (98–135)
UD		180.5 (160–210)	46.0 (40–48)	25.0 (18–32)	125.0 (106–146)

*P* value		0.3864	0.6236	0.3240	0.4412

NC	6 weeks	186.5 (178–202)	54.5 (45–60)	17.5 (13–26)	122.5 (104–132)
UD		188.5 (180–197)	50.5 (47–60)	18.5 (16–26)	127.0 (110–140)

*P* value		0.6842	0.7394	0.4359	0.4813

NC	14 weeks	189.0 (186–200)	55.5 (48–60)	18.0 (16–20)	121.0 (119–140)
UD		192.5 (182–204)	53.0 (49–58)	20.0 (18–24)	127.5 (118–132)

*P* value		0.8857	0.6857	0.2000	0.8857

NC	26 weeks	191.0 (188–195)	52.5 (46–60)	18.5 (16–22)	133.0 (125–142)
UD		199.0 (186–215)	47.0 (42–50)	23.0 (19–24)	135.0 (122–152)

*P* value		0.3429	0.2000	0.1143	0.8857

NC: nondiabetic control; UD: untreated diabetic.

NC = UD at 2, 6, 14, and 26 weeks of follow-up (Mann-Whitney test).

**Table 3:** Mean ± SD of fresh liver weight (g) and fresh liver weight/body weight ratio of nondiabetic control rats and untreated diabetic rats during the various experimental follow-up periods.

Groups	Parameters and follow-up					
	Fresh liver weight (g)			Fresh liver weight/body weight ratio		
	6 weeks	14 weeks	26 weeks	6 weeks	14 weeks	26 weeks
NC	11.1420 ± 1.6050	12.3300 ± 1.0080	12.3320 ± 2.1700	0.0445 ± 0.0036	0.0277 ± 0.0041	0.0243 ± 0.0034
UD	10.8990 ± 1.0430	9.5560 ± 1.0430	11.5040 ± 0.5772	0.0463 ± 0.0041	0.0555^*^ ± 0.0128	0.0595^*^ ± 0.0104

*P* value	0.1076	0.0865	0.3075	0.3064	<0.0001	<0.0001

NC: nondiabetic control; UD: untreated diabetic.

^*^NC < UD at 14 and 26 weeks of follow-up (Student's *t*-test).
